# Liang-Ge-San: a classic traditional Chinese medicine formula, attenuates acute inflammation via targeting GSK3β

**DOI:** 10.3389/fphar.2023.1181319

**Published:** 2023-06-29

**Authors:** Liling Yang, Lijun Yan, Weifu Tan, Xiangjun Zhou, Guangli Yang, Jingtao Yu, Zibin Lu, Yong Liu, Liyi Zou, Wei Li, Linzhong Yu

**Affiliations:** ^1^ Department of Pharmacy, The Binhaiwan Central Hospital of Dongguan, The Dongguan Affiliated Hospital of Medical College of Jinan University, Dongguan, China; ^2^ Third Level Research Laboratory of State Administration of Traditional Chinese Medicine, School of Traditional Chinese Medicine, Southern Medical University, Guangzhou, China; ^3^ Department of Neonatology, The Binhaiwan Central Hospital of Dongguan, The Dongguan Affiliated Hospital of Medical College of Jinan University, Dongguan, China; ^4^ Guangdong Provincial Key Laboratory of Research and Development of Natural Drugs, School of Pharmacy, Guangdong Medical University, Dongguan, China; ^5^ Department of Central Laboratory, The Binhaiwan Central Hospital of Dongguan, The Dongguan Affiliated Hospital of Medical College of Jinan University, Dongguan, China

**Keywords:** Liang-Ge-San, traditional Chinese medicine, sepsis, acute inflammation, LPS, GSK-3β, macrophage, immunomodulatory

## Abstract

Sepsis is a serious life-threatening health disorder with high morbidity and mortality rates that burden the world, but there is still a lack of more effective and reliable drug treatment. Liang-Ge-San (LGS) has been shown to have anti-inflammatory effects and is a promising candidate for the treatment of sepsis. However, the anti-sepsis mechanism of LGS has still not been elucidated. In this study, a set of genes related to inflammatory chemotaxis pathways was downloaded from Encyclopedia of Genes and Genomes (KEGG) and integrated with sepsis patient information from the Gene Expression Omnibus (GEO) database to perform differential gene expression analysis. Glycogen synthase kinase-3β (GSK-3β) was found to be the feature gene after these important genes were examined using the three algorithms Random Forest, support vector machine recursive feature elimination (SVM-REF), and least absolute shrinkage and selection operator (LASSO), and then intersected with possible treatment targets of LGS found through the search. Upon evaluation, the receiver operating characteristic (ROC) curve of GSK-3β indicated an important role in the pathogenesis of sepsis. Immune cell infiltration analysis suggested that GSK-3β expression was associated with a variety of immune cells, including neutrophils and monocytes. Next, lipopolysaccharide (LPS)-induced zebrafish inflammation model and macrophage inflammation model was used to validate the mechanism of LGS. We found that LGS could protect zebrafish against a lethal challenge with LPS by down-regulating GSK-3β mRNA expression in a dose-dependent manner, as indicated by a decreased neutrophils infiltration and reduction of inflammatory damage. The upregulated mRNA expression of GSK-3β in LPS-induced stimulated RAW 264.7 cells also showed the same tendency of depression by LGS. Critically, LGS could induce M1 macrophage polarization to M2 through promoting GSK-3β inactivation of phosphorylation. Taken together, we initially showed that anti-septic effects of LGS is related to the inhibition on GSK-3β, both *in vitro* and *in vivo*.

## Introduction

Sepsis is a fatal immunological sickness characterized by a strong inflammatory response that cannot be managed, immunosuppression that develops later in the illness, and tissue destruction. By activating different immune cells, lipopolysaccharide (LPS), the primary cause of gram-negative bacterial infections, can cause excessive inflammatory responses that contribute to sepsis and septic shock. As a result, LPS is frequently used in sepsis models. At present, there is no specific effective treatment for LPS-triggered sepsis, and the use of glucocorticoids has been widely questioned (Yang J.-G. et al., 2019). Despite the application of novel nano antibiotics, LPS adsorbed hemophiliacs (oXiris), mechanical ventilation and other comprehensive therapeutic measures, the case fatality rate remains high. The latest epidemiological data show that: Globally, nearly 49 million people suffer from sepsis each year and over 11 million deaths are linked to sepsis, accounting for 20% of all annual deaths. In China, the mortality rate of sepsis amounts to 66.7%, and about 75% of the survivors has post-sepsis syndrome (Rittirsch et al., 2008; Yamamoto et al., 2011; Huang et al., 2020; Liu et al., 2021). Therefore, it is important to search for multiple drugs that block or inhibit the LPS inflammatory cascade.

Neutrophils and macrophages are the target immune cells of LPS and play an important role in the development and progression of LPS infectious diseases. On the one hand, inflammatory cells such as macrophages and neutrophils will quickly migrate to the site of infection to phagocytic and degrade pathogens by releasing pro-inflammatory cytokines and chemokines to maintain the homeostasis of the body (Serhan et al., 2007; Granger et al., 2010; Kolaczkowska and Kubes, 2013; Poon et al., 2014). On the other hand, when the homeostasis is damaged by massive or continuous external stimulation, activated macrophages become overly M1-polarized, killing target cells and damaging tissues. In the late stage of infection, macrophages become M2-polarized and participate in immunosuppression, inducing secondary infection. Meantime, highly activated neutrophils can also play a variety of pro-inflammatory effects by releasing dissolved trapping nets and reactive oxygen species, further aggravating tissue dysfunction and injury (Kreuger and Phillipson, 2016; Potey et al., 2019). Therefore, inhibition of excessive infiltration and polarization of leukocytes is of great importance in alleviating LPS-induced excessive inflammation.

It is worth noting that chemokines and their receptors are key factors in the regulation of normal immunity of the body and the recruitment of inflammatory cells in numerous inflammatory diseases (Stone et al., 2017). The occurrence of microbial infections often stimulates cells to send out powerful chemokine signals, which help the body to generate a complex immune response to control the growth of invading pathogens (Skinner et al., 2019). In particular, the migration and function of neutrophils cannot be achieved without the combined coordination of extracellular signals such as chemokines and important components of neutrophils such as chemokine receptors (Liu Y. et al., 2022). Thus, inflammatory cells and chemotaxis are interdependent and inseparable. However, the potential biological targets that determine the migratory capacity of neutrophils have not been fully elucidated. Therefore, this study will explore possible biological targets in sepsis based on inflammatory chemotactic pathways that may affect the function of inflammatory cells.

A large number of clinical observations show that traditional Chinese medicine (TCM) has advantages and characteristics in regulating the inflammatory response, which has attracted considerable attention in the treatment of sepsis, acute respiratory (ARDS) and the current epidemic corona virus disease 2019 (COVID-19). It has been advocated by the China National Health Commission for the diagnosis and treatment plan of LPS inflammatory diseases including sepsis, and the research on the function and mechanism of association medications has drawn attention (Cao et al., 2019; Ding et al., 2020; Fan et al., 2020). Liang-Ge-San (LGS), a well-known TCM formula, was first mentioned in the book “Taiping Huimin Heji Jufang” over a thousand years ago. Since then, it has also been included in numerous editions of the textbook “Fangjixue”. LGS is generally used to treat infections in clinic, including pharyngitis, tonsillitis, sepsis and acute lung injury (ALI) (Lee et al., 2017). Some clinical studies have shown that LGS could protect patients against sepsis in a plus-minus therapy, as indicated by a decreased body temperature, improvement of oxygenation index level, reduction of pro-inflammatory factors release, depression of APACHE Ⅱ score and increase of survival rate (Su, 2011; Liu, 2014; Du, 2019; Li et al., 2020; Qiao et al., 2020). In our previous study, we have demonstrated that LGS suppressed LPS-induced ALI in mice by activating the cholinergic anti-inflammatory pathway, up-regulating the expression of the miR-21 and protected zebrafish against LPS-induced death which was related to the inhibition on p-JNK and p-Nur77 (Liu et al., 2016; Yang H. et al., 2019; Zhou et al., 2020; Lu et al., 2021). However, as a well-known prescription for the treatment of febrile diseases, the mechanism of LGS is also multiple. Based on our previous research, whether other modulators are associated with anti-acute inflammatory effects of LGS needs further explored. In this study, we performed in-depth exploration in underlying molecular mechanism related to anti-inflammatory chemotactic effects of LGS.

In this study, R language was utilized for statistical analysis of two separate sepsis gene expression profile data sets in the U.S. Gene Expression Omnibus (GEO) database, and the possible differentially expressed genes were first obtained through differential analysis. Then the support vector machine recursive feature elimination (SVM-RFE), random forest (RF) and the least absolute shrinkage and selection operator (LASSO) were used to identify the signature genes. Potential drug targets of LGS were collected and combined with the characteristic genius of sepsis, and finally the signature genes related to LGS were screened. In order to further verify signature genes, operating characteristic curves (ROC) analysis and clinical correlation analysis were used to evaluate the predictive effect of signature genes. In addition, we studied the level of immune cell infiltration in the sepsis group, and finally calculated the relationship between signature genes and immunity. This study sought to identify potential LGS targets for sepsis linked with inflammatory infiltration by genetic screening. Finally, we screened out the signature gene GSK-3β, which is associated with inflammatory chemotaxis in sepsis, and demonstrated that the anti-septic effects of LGS *in vitro* and *in vivo* through meddling in GSK-3 β expression to regulate leukocytes migration and polarization.

## Materials and methods

### Data processing and download of the sepsis dataset

Retrieved and downloaded sepsis gene expression profile data from the GEO database (https://www.ncbi.nlm.nih.gov/geo/) from its inception to 01 November 2022. The following filtering criteria were applied: 1) Disease type “sepsis”; 2) Tissue source “blood”; 3) Entry type “Series "; 4) Study type " Expression profiling by array”; 5) Top Organisms were selected as “*Homo sapiens*”; 6) The sample set contained disease and control groups. Two gene expression profile datasets, GSE13904 (Wong et al., 2009) and GSE4607 (Wong et al., 2008), were finally obtained. The Kyoto Encyclopedia of Genes and Genomes (KEGG, https://www.kegg.jp/) was searched for chemokine signaling pathway (map04062) and pathway-related genes were extracted. The expression profile dataset obtained from GEO was cross-platform normalized by the “ComBat” R package to remove batch effects, and the expression data matrix containing only inflammatory chemotaxis-related genes was extracted after intersection with pathway-related genes (Zhang et al., 2020). To study differences in gene expression, the “Limma” R package was utilized, and particular up- and downregulated genes were selected (Ritchie et al., 2015). Among them, disease genes that met the pre-defined adjusted *p*-value < 0.05 and log FC absolute value >0.3 were differentially expressed genes. Then, these genes were displayed as a heat map using the “Heatmap” R package and as a volcanic map using the “ggplot2” R package.

### Enrichment of functionality and pathway

Functional enrichment of possible differentially expressed genes was performed to explore the possible functions and signaling pathways of potential targets. Gene Ontology (GO) is a method to uncover the biological functions of genes, especially in molecular functions (MF), biological processes (BP) and cellular components (CC). KEGG enrichment analysis can be used to analyze gene functions and related high-level genomic functional information. Investigating the GO function of differentially expressed genes and extracting KEGG signaling pathway data of these genes by using the “GOplot” and “cluster profiler” R packages will help us better understand the biological functions and potential processes of target genes (Walter et al., 2015).

### Acquisition of LGS pharmacodynamic targets

Traditional Chinese medicine systems pharmacology database and analysis platform (TCMSP, https://old.tcmsp-e.com/tcmsp.php) is able to capture the relationship between drug, target and disease and is used to find possible targets of action of drug candidates (Ru et al., 2014). Encyclopedia of Traditional Chinese Medicine (ETCM, http://www.nrc.ac.cn:9090/ETCM/) also provides predictive target genes for herbal ingredients, herbs and formulations to facilitate functional and mechanistic studies of herbal medicines (Xu et al., 2019). The chemical components of all LGS formulations were screened from TCMSP and ETCM. The following pre-conditions were met: oral bioavailability (OB) ≥ 30% and drug-likeness (DL) ≥ 0.18 for LGS active ingredients and their targets were extracted.

### Signature gene screening

The differentially expressed genes obtained above were screened to isolate signature genes for the diagnosis of sepsis. SVM is a classifier-building algorithm that creates a decision boundary between two categories and is able to predict labels from one or more feature vectors (Noble, 2006). SVM-RFE achieves higher classification performance by filtering relevant features and removing relatively unimportant feature variables (Huang et al., 2014). One of the most used algorithms, LASSO, is also employed in clinical decision making (Kang et al., 2021). LASSO was implemented via “glmnet” R package. The binomial distribution variables are then used for LASSO classification, plus a minimum criterion (1-SE criterion) based on the standard error λ values, which are used to build the model (Zhao et al., 2022). RF is an ensemble learning algorithm based on decision trees, which can build multiple decision tree models from a random sample of the data set to make predictions together (Svetnik et al., 2003). This study used the above three machine learning algorithms in order to identify signature genes. Then intersected with potential drug targets of the LGS using the “Venn” R package to obtain the final signature genes. In addition, to screen for signature genes and assess their diagnostic efficiency, the R package was used to create subject ROC to determine the area under the curve (AUC) (Robin et al., 2011). An AUC greater than 0.7 indicated favorable diagnostic performance.

### Immune cell infiltration

CIBERSORT allows the analysis of the expression matrix of human immune cell subtypes and can be used to detect differences in immune cells between septic patients and healthy individuals (Newman et al., 2015). In this study, the GEO dataset (GSE13904 and GSE4607) was utilized to screen for cells with significant changes in immune cell infiltration between sepsis and normal populations. At same time, we used the “corrplot” R package to calculate the correlation between immune cell infiltration and signature genes.

## Experimental verification

### Experimental materials

Forsythia suspensa (Thunb.) Vahl (Lot: NO.211201101; origin: Shanxi Province, China), Rheum palmatum L. (Lot: NO.211100199; origin: Sichuan Province, China), Scutellaria baicalensis Georgi (Lot: NO.211002401; origin: Hebei Province, China), Gardenia jasminoides J. Ellis (Lot: NO.211002521; origin: Jiangxi Province, China), Glycyrrhiza uralensis Fisch. ex. DC. (Lot: NO.210902241; origin: Neimenggu Province, China), Mentha canadensis L. (Lot: NO.211000191; origin: Jiangsu Province, China), Sodium sulfate (Lot: NO.210902001; origin: Jiangsu Province, China) were obtained from Kangmei (Guangzhou Province, China). Anti-Mannose Receptor antibodies were purchased from Abcam. iNOS (D6B6S) Rabbit mAb, GSK-3β (D5C5Z) XP^®^ Rabbit mAb and Phospho-GSK-3β (Ser9) (D85E12) XP^®^ Rabbit mAb antibodies were purchased from Cell Signaling Technology. TRIzol was purchased from Invitrogen. PrimeScript Tyragent Kit with gDNA Eraser and SYBR^®^ Premix Ex TaqTM II were purchased from Takara Corporation, Japan. 055: B5) were purchased from Sigma-Aldrich. Fetal bovine serum, high sugar medium, and penicillin/streptomycin were purchased from GIBCO (Grand Island, United States). Hematoxylin and eosin were obtained from Yuanye Biotech (Shanghai, China). Dexamethasone (DEX) was obtained from Tianxin (Guangzhou, China). TWS119 and Wortmannin were purchased from Abmole (Houston, United States). BCA protein assay kit and enhanced chemiluminescence (ECL) kit were purchased from Thermo Fisher Scientific (Waltham, United States). Lipopolysaccharide (LPS,055: B5), Thiazolyl Blue Tetrazolium Bromide (MTT), DMSO, Paraffin, and other reagents were obtained from Sigma-Aldrich (St. Louis, United States).

### Preparation of Chinese herbal extracts and quality control

All Chinese medicinal materials including Forsythia suspensa (Thunb.) Vahl, Rheum palmatum L., Scutellaria baicalensis Georgi., Gardenia jasminoides Ellis, Glycyrrhiza uralensis Fisch., Mentha haplocalyx Briq. and Natrii Sulfas. were authenticated by Prof. Ji Ma (Southern Medical University). All of herbs contained in the formula for LGS were provided by Kangmei (Guangzhou, China). The quality control and the origin of each herb have been performed in accordance with Chinese Pharmacopoeia (2015 Edition). The preparation and high-performance liquid chromatography (HPLC) fingerprint analysis of LGS was performed according to our previous study (Yang H. et al., 2019; Zhou et al., 2020).

The preparation of LGS: Firstly, according to the recipe LGS (Table 1), herbs were weighed and soaked with 10-time volume of water for 30 min. Then, the first herbs were decocted for 10 min. Afterwards, the second herbs were added to decoction for another 10 min. Subsequently, the recipe LGS was decocted again with another 6-time volume of water. Finally, the last batch of herb was added to the aqueous extract. The extract was pooled and further concentrated to 200 mL. Concentrated extract was lyophilized into powder and stored in desiccators (Yang H. et al., 2019; Zhou et al., 2020).

**TABLE 1:** Main composition and decoction order of LGS.

Main composition	Latin scientific name	Amount (g)	Decoction order
Fructus forsythiae (Lian Qiao)	*Forsythia suspense* (Thunb.) *Vahl*	24	First batch
Fructus Gardeniae (Zhi Zi)	*Gardenia jasminoides* Ellis	6	First batch
Radix Scutellariae (Huang Qin)	*Scutellaria baicalensis* Georgi	6	First batch
Liquorice (Gan Cao)	*Glycyrrhiza uralensis* Fisch	12	First batch
Rheum officinale (Da Huang)	*Rheum palmatum* L	12	Second Batch
Mint (Bo He)	*Mentha haplocalyx* Briq	6	Second Batch
Mirabilite (Mang Xiao)	*Natrii Sulfas*	12	Last Batch

Quality Control of LGS (HPLC fingerprint analysis): Using HPLC (Agilent 1260, United States) to analyze for specific ingredients and chemical fingerprints as well as quantification of marker compounds of LGS. Chromatographic column: Zorbax Eclipse XDB-C18 column (250 × 4.6 mm, 5 μm, Agilent, United States); Detector: Infinity VL diode array detector (G1315D, Agilent, United States); Mobile phase system: A/B = water with 0.1% phosphoric acid/methanol; Column temperature: 38°C; Flow rate: 1 mL/min; Detection wavelength: 235 nm. The chemical fingerprints and the quantification of marker compounds of LGS had the similar chemical profile to our previous fingerprinting (Hu et al., 2014; Yang H. et al., 2019; Zhou et al., 2020).

### Experimental animals

Zebrafish were housed in a zebrafish circulatory system (28.5°C) with a diurnal cycle time of 14/10 h. Zebrafish embryos were collected and reared according to the conditions described by Westerfield (Westerfield, 2007). Zebrafish were provided by the Laboratory of Pharmacology of Traditional Chinese Medicine, Southern Medical University. All experimental protocols and procedures were approved by the Animal Experimental Ethics Committee of Southern Medical University.

### Experimental cells

The mouse peritoneal mononuclear macrophage cell line RAW264.7 purchased from American Type Culture Collection was cultured in DMEM complete medium containing 10% (V/V) fetal bovine serum and 0.5% penicillin/streptomycin at 37°C in a 5% CO_2_ incubator.

### Construction of zebrafish inflammation model

A model of endotoxin infection was established by microinjection of 2 nL LPS (0.5 mg/mL) into zebrafish yolk 3 days post-fertilization (3dpf) (Yang et al., 2014), with PBS serving as a negative control. Juvenile fish at the end of injection were transferred to 6-well plates after recovery from anesthesia (0.02% tricaine), and treatment groups were given LGS or TWS119 or Wortmannin or DEX and incubated at 28.5°C.

### Observation of neutrophil recruitment and assessment inflammatory criteria in zebrafish

Transgenic line Tg (MPO: GFP) *in vivo* neutrophils have green fluorescent markers, which enable *in vivo* tracing of neutrophils. Zebrafish were collected 12hpi after drug administration, and the recruitment of neutrophils in the yolk sac after drug treatment was observed by body fluorescence microscopy (Olympus MVX10). Assessment of neutrophil recruitment and grading of inflammation in the zebrafish yolk sacs. And the assessment criteria are as follow: Grade 1 = normal (none or several neutrophils, no necrosis); Grade 2 = neutrophils scattered in the yolk, no necrosis; Grade 3 = neutrophils recruitment to the LPS site, no necrosis; Grade 4 = bulk infiltration of neutrophils or neutrophils adhesion, no necrosis; Grade 5 = yolk deformation and necrosis (Yang et al., 2017).

### Histopathological observations

Zebrafish yolk sacs were fixed using 4% (v/v) paraformaldehyde and embedded in paraffin for 12 hpi after administration, sectioned for 5 μm, dewaxed and stained with H&E, and observed for yolk sac under a light microscope (IX53, Olympus, Tokyo, Japan).

### RAW264.7 cellular activity assay

Cell viability was determined by 3-[4,5-dimethylthiazol-2-yl]-2,5-diphenyltetrazoliumbromide (MTT) assay as described previously (Lu et al., 2018). RAW264.7 cells were inoculated into 96-well plates at a density of 8×10^3^ cells per well and incubated for 24 h. Subsequently, cells were treated with different concentrations of LGS (12.5, 25, 50, 100, 200, 400, 800 μg/mL) or GSK-3β inhibitor TWS119 (2.5, 5, 6.25, 12.5, 25, 50 μM) for 24 h followed by incubating with 30 μL of MTT (5 mg/mL) per well for another 4 h at 37 °C. The supernatants were removed and 100 μL of DMSO was added to dissolve the formazan crystals. The absorbance at 490 nm was measured using a microplate reader (Thermo Fisher Scientific, United States).

### Western blot analysis

RAW264.7 cells were inoculated at a density of 5 × 10^5^ cells in 60 mm culture dishes. RAW264.7 cells (M0 type macrophages) were pre-stimulated with 100 ng/mL LPS for 48 h to obtain M1 type macrophages. LGS (62.5, 125, 250 μg/mL) or TWS119 (5 μM) was co-stimulated with LPS (1 μg/mL) for 48 h and the cells were lysed using lysis buffer 2 to obtain cellular whole proteins. The protein concentration was measured by the BCA method.

### QPCR

After extraction of total RNA using the miRcute miRNA kit, the quality and quantity of RNA were detected using a DS-11+ spectrophotometer (Denovix). After reverse transcription of RNA using the PrimeScriptTM RT reagent Kit with gDNA Eraser kit, the relative expression levels of relevant genes were detected using SYBR^®^ Premix Ex TaqTM II (Tli RNaseH Plus) with β-actin as an internal reference. All qPCR reactions were performed using the LightCycler^®^ 96 Real-Time PCR System (Roche) and relative gene expression was calculated using 2^−ΔΔCt^.The primer sequences used are shown in Table 2.

**TABLE 2:** Specific primer sequences.

Gene	Genus	Forward primers (5′-3′)	Reverse primers (5′-3′)
GSK-3β	Mouse	GAC​AGT​GGT​GTG​GAT​CAG​TTG​GTG	AAT​GTC​CTG​CTC​CTG​GTG​AGT​CC
	zebrafish	GAC​CGC​TCG​ACT​GAC​TCC​TCT​C	GTT​GAA​GCT​CCT​GCC​TGG​TTA​CG
MR	Mouse	ACC​TGG​CAA​GTA​TCC​ACA​GCA​TTG	GCA​GTC​CTC​CTG​TCT​GTT​GTT​CTC
iNOS	Mouse	TGC​CAC​GGA​CGA​GAC​GGA​TAG	CTC​TTC​AAG​CAC​CTC​CAG​GAA​CG
β-actin	Mouse	ATG​TGG​ATC​AGC​AAG​CAG​G	GTC​AAA​GAA​AGG​GTG​TAA​AAC​G
	zebrafish	ATG​GAT​GAG​GAA​ATC​GCT​G	ATG​CCA​ACC​ATC​ACT​CCC​TG

### Statistical analysis

All data were expressed as mean ± standard deviation (Mean ± SD). In addition, SPSS 20.0 software ANOVA was used to assess the differences between multiple groups, and the Turkey *post hoc* test was used for the *post hoc* test. *p* < 0.05 indicates that the differences are statistically significant.

## Result

### Inflammatory chemotaxis differentially expressed genes

**FIGURE 1:**
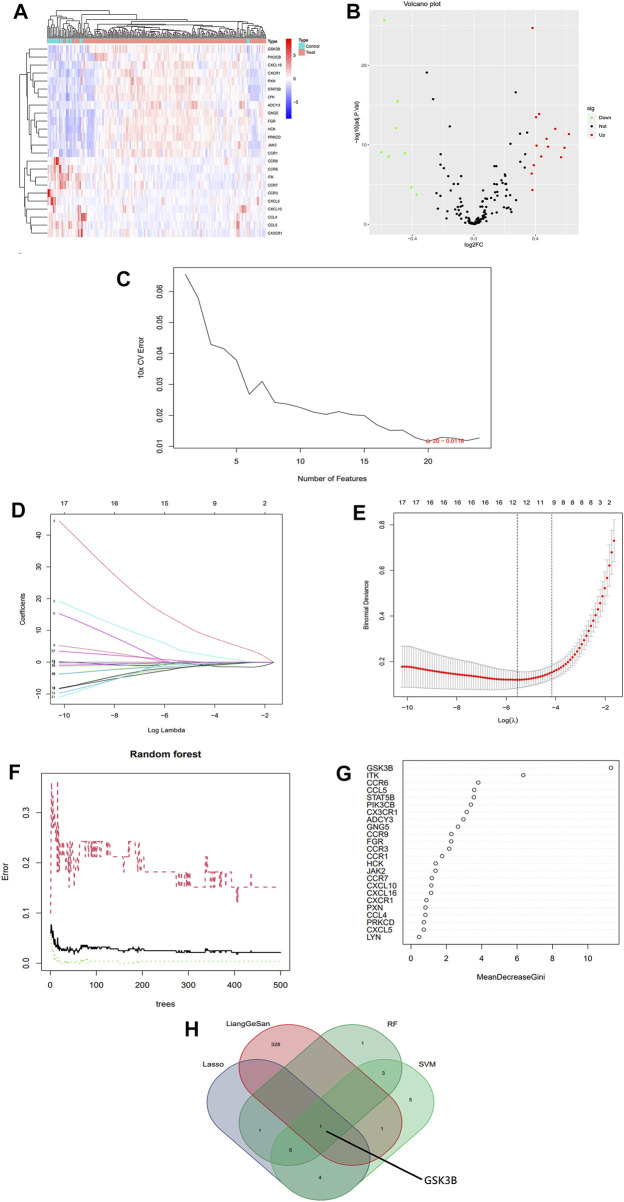
Bioinformatics screening for sepsis signature genes. **(A)** Heat map of differential expression analysis of gene expression profile data. **(B)** Volcano map of differentially expressed genes associated with inflammatory chemotaxis in sepsis. **(C)** Selection of signature biomarker genes by support vector machine recursive feature elimination (SVM-RFE). **(D,E)** Adjustment of feature selection in the minimum absolute shrinkage and selection operator model (LASSO). **(F,G)** Relationship between Random Forest error rate and number of classification trees and the most meaningful signature genes. **(H)** Venn diagram of the intersection of three machines learning algorithms and LGS drug targets, with results showing GSK-3β as the final signature gene.

Two gene expressions profiling datasets, GSE13904 and GSE4607, were obtained from the GEO database, and 279 samples were finally obtained for inclusion in the subsequent differential gene expression analysis, including 246 sepsis patients and 33 normal controls. 178 relevant genes were obtained by KEGG search of the inflammatory chemotaxis pathway. After eliminating the batch effect, gene expression differential analysis screening was performed to obtain 24 inflammatory chemotaxes differentially expressed genes. Bioinformatic screening of the signature genes is shown in Figure 1. The intersection of the candidate genes obtained by each of the three different machine learning algorithms was used to identify GSK-3β as the final signature gene.

### Functional analysis of critical genes

**FIGURE 2:**
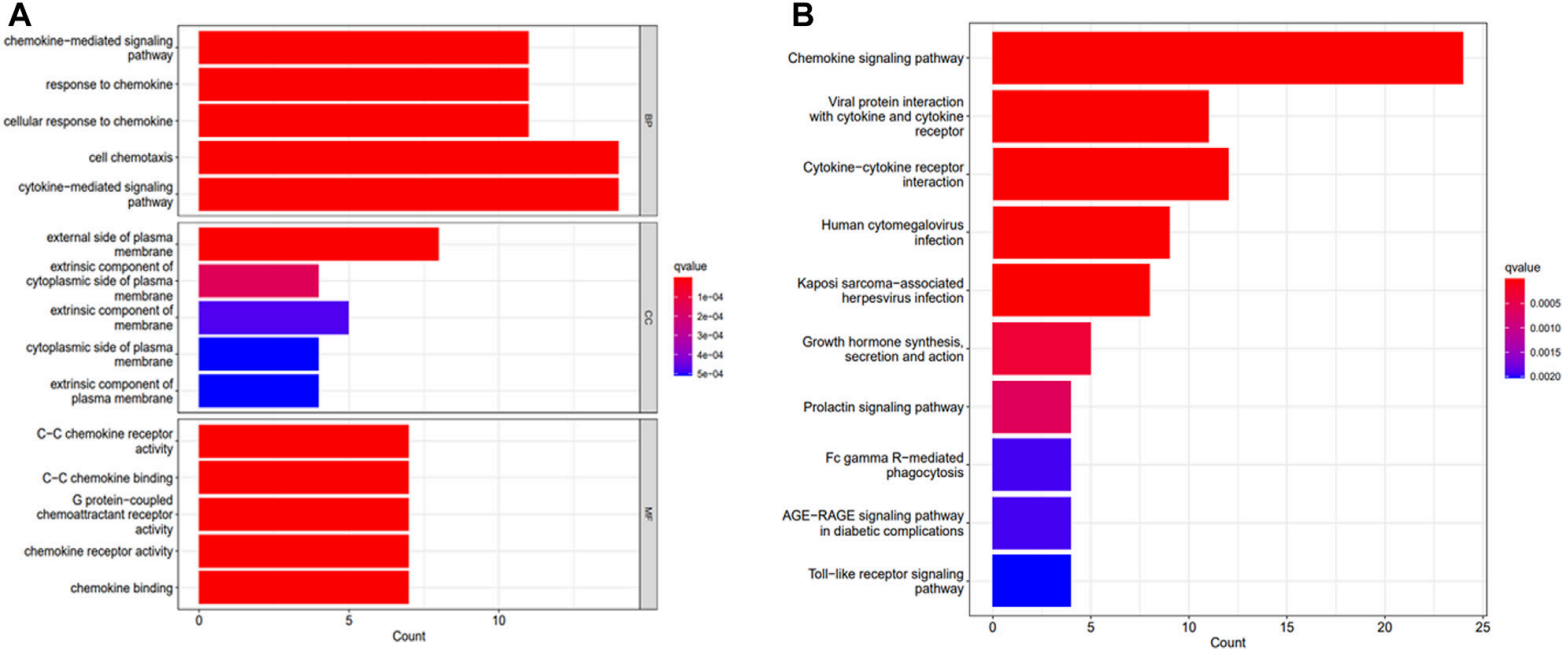
Functional analysis of DEGs. **(A)** GO analysis. **(B)** KEGG analysis.

All DEGs were functionally enriched, and 5 GO keywords were exhibited in the GO bar plot according to *p* < 0.05 (Figure 2A; Supplementary Table S1). The results showed that the enrichment of biological processes (BP) was mainly related to chemokine-mediated signaling pathways, cellular responses to chemokines, and cellular chemotaxis. Molecular functions (MF) were related to chemokine receptor activity and chemokine binding such as C- C chemokine receptor and G protein-coupled chemokine receptor. The cellular component (CC) related to the outer side of the plasma membrane. Results of KEGG analysis showed an association with chemokine signaling pathways, viral protein-cytokine and cytokine receptor interactions, cytokine-cytokine receptor interactions, human cytomegalovirus infection and Kaposi’s sarcoma-associated herpesvirus infection (Figure 2B; Supplementary Table S2).

### Active ingredients and targets of LGS

According to the set screening conditions, a total of 274 candidate active compounds were collected from the TCMSP and TCMIP databases, including 81 from Rheum palmatum L., 23 from Forsythia suspensa (Thunb.) Vahl, 36 from Scutellaria baicalensis Georgi., 92 from Glycyrrhiza uralensis Fisch. ex. DC., 15 from Gardenia jasminoides J. Ellis, 22 from Mentha canadensis L., 1 from Sodium sulfate and 4 from Lophatherum gracile Brongn., and a total of 330 corresponding targets were extracted after deleting duplicate active ingredients (Figure 1H).

### Selection of signature genes

Three machine algorithms were used to identify feature genes: SVM - RFE to filter 20 signature genes (Figure 1C; Supplementary Table S2); LASSO regression analysis to select 12 predicted genes from statistically significant univariates (Figures 1D, E; Supplementary Table S2); and RF combined with feature selection to determine the relationship between 12 genes of relative importance (Figures 1F–G; Supplementary Table S2). One overlapping gene GSK-3β was found using a Venn diagram intersection to visualize the signature genes obtained from the above three machine learning classification methods as well as the LGS potential targets (Figure 1H).

**FIGURE 3:**
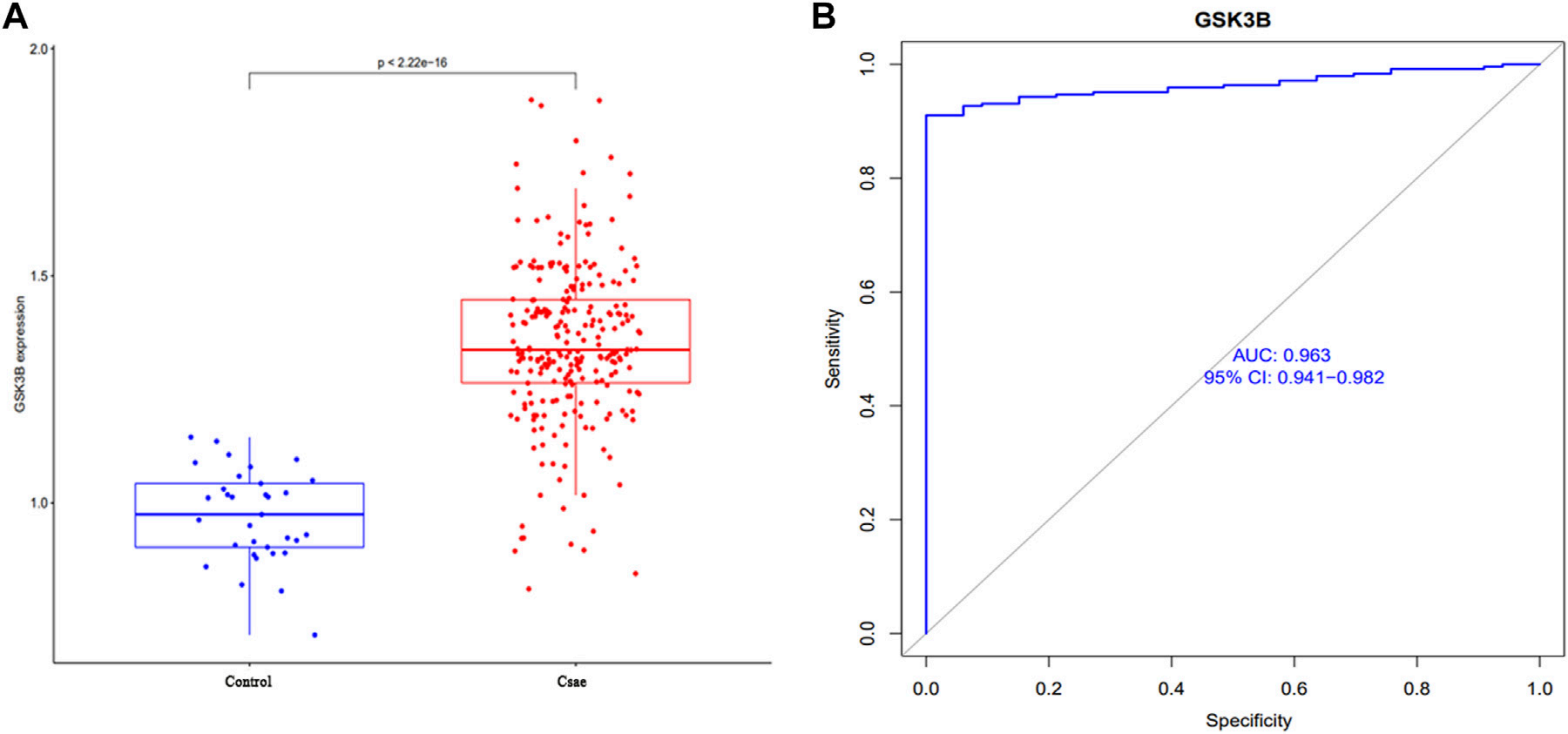
Analysis of clinical correlation between GSK-3β expression and sepsis **(A)** and the ROC curve to assess the predictive performance **(B)**.

As shown in Figure 3, GSK-3β gene expression data was extracted and analyzed with clinical grouping information, and the results suggested that GSK-3β expression was significantly upregulated in the sepsis group, with statistically significant differences (*p* < 0.001). In addition, the AUC result was 0.963 (95% CI 0.941–0.982), suggesting that GSK-3β has superior performance in predicting sepsis and can be used as a diagnostic marker.

### Immune cell infiltration

**FIGURE 4:**
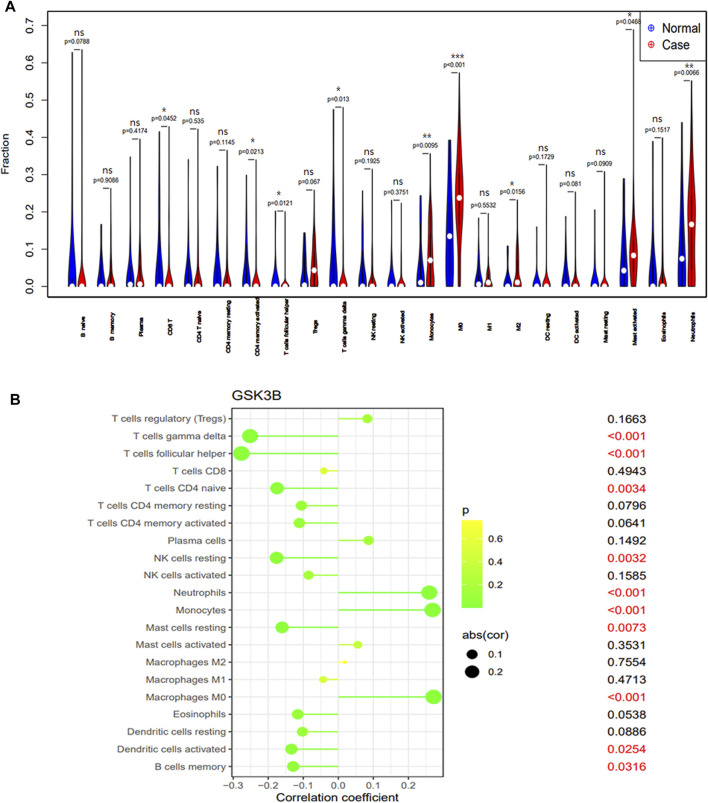
The immune cell infiltration association with signature genes. **(A)** The immune cell infiltration between the sepsis patient and healthy cohort. **(B)** The association between the signature gene GSK-3β and significantly different immune cell infiltration. “ns” means *p* ≥ 0.05, ^*^ *p* < 0.05, ^**^ *p* < 0.01, and ^***^ *p* < 0.001.

Immunological characteristics of different populations were evaluated according to immune cell infiltration. Compared with normal cohort, patients with sepsis have higher monocytes, M0 macrophages, M2 macrophages, activated mast cells, neutrophils infiltration and lower CD8^+^ T cell, memory activity CD4^+^ T cell, follicular helper T cells, gamma delta T cells, activated dendritic cell infiltration (Figure 4A). Expression of the signature gene GSK-3β were negatively correlated with the infiltration of gamma delta T cells, follicular helper T cells, naive CD4^+^T cell, resting NK cells, resting mast cells, activated dendritic cell, memory B cell and positively correlated with the infiltration of neutrophils and monocytes (Figure 4B).

### LGS Inhibits LPS-induced Neutrophil Inflammatory Infiltration in Zebrafish by Suppressing GSK-3β Expression

**FIGURE 5:**
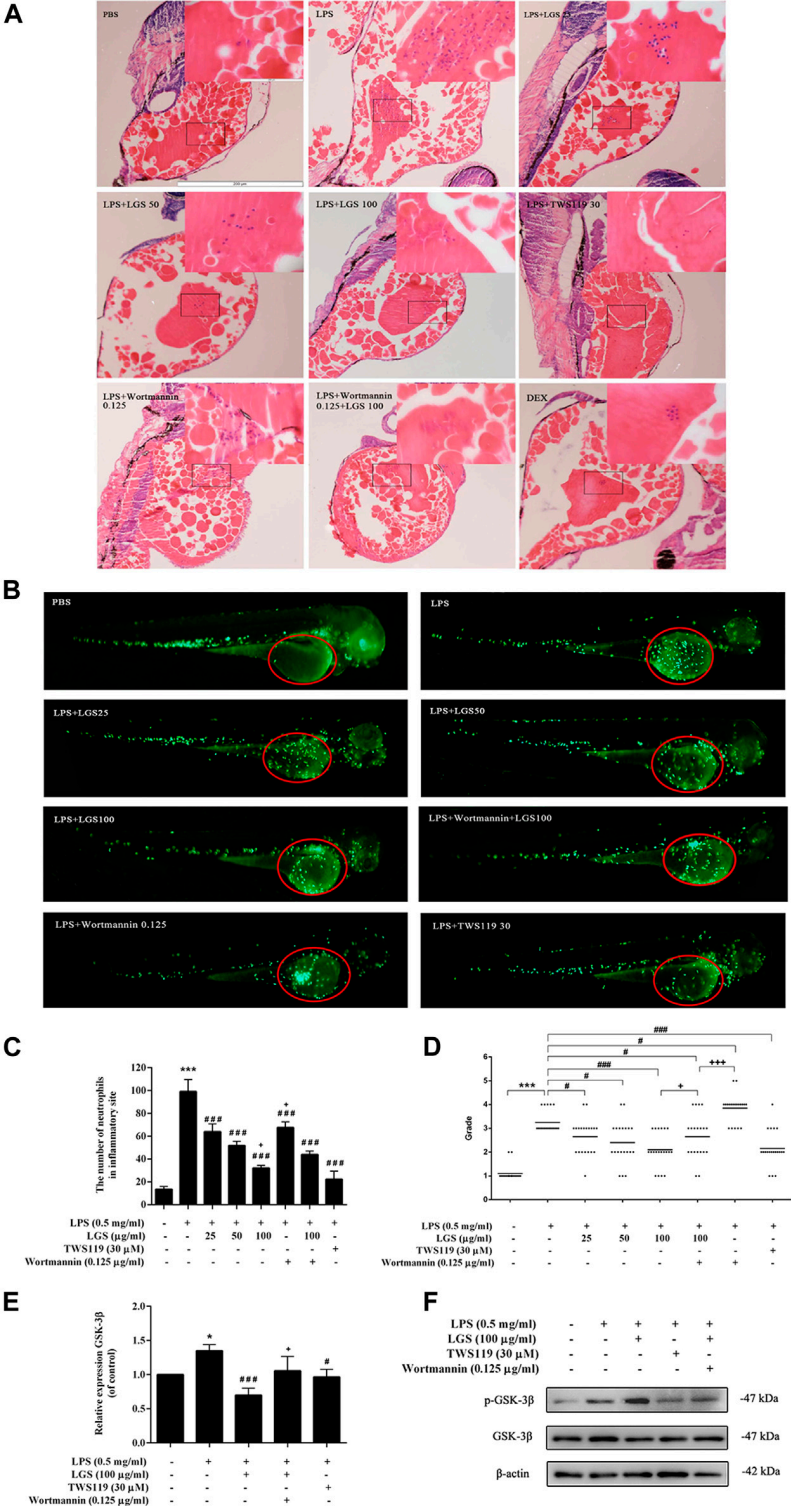
LGS inhibits LPS-induced neutrophil inflammatory infiltration by suppressing GSK-3β expression in zebrafish. 3dpf larvae yolk-microinjected with 0.5 mg/mL LPS or PBS, and then treated with DEX (5 μg/mL) or LGS (25, 50, 100 μg/mL) or TWS119 (30 μM) or Wortmannin (0.125 μg/mL). After 12 hpi, larvae were dehydrated and embedded in paraffin for H&E staining assay **(A)**, ×100); Representative fluorescence images **(B)**, red circle), Statistical analysis **(C)** and inflammatory grading scatter diagrams **(D)** of neutrophils in yolk were shown; The mRNA expression level of GSK-3β was determined by q-PCR **(E)** The protein expressions of p-GSK-3β (Ser9) and GSK-3β were measured by Western blot **(F)**. Data are presented as mean ± SEM, n = 3, LPS group vs. blank group, ^*^ *p* < 0.05; vs. LPS group, ^###^ *p* < 0.0001, ^#^ *p* < 0.05; vs. LPS + LGS group, ^+^ *p* < 0.05.

Neutrophil chemotaxis and recruitment are essential for the inflammatory response, and this process are regulated by the central inflammatory response kinase GSK-3β (Chen et al., 2020). To investigate whether LGS inhibits inflammatory cell infiltration by affecting GSK-3β signaling, we used TWS119 (GSK-3β inhibitor) and Wortmannin (GSK-3β agonist) to block or activate GSK-3β, respectively. HE staining revealed that both LGS and TWS119 treatment reduced yolk congestion, inflammatory cell infiltration and improved pathological conditions due to LPS. The increase in yolk inflammatory cell infiltration after the addition of Wortmannin treatment compared to LGS treatment alone (Figure 5A). Then, we used a transgenic line Tg (MPO: GFP) to observe the neutrophils recruitment. At 3.5 dpf, neutrophils mainly appeared in the PBI, and the head stroma and epidermis both expressed a few of these cells (Le Guyader et al., 2008). As showed in Figures 5B, C, at 12hpi, yolk with severe neutrophils recruitment were observed in LPS group, but only a small amount of sparsely distributed neutrophils in LGS (25, 50, 100 μg/mL) or TWS119 (30 μM) treated larvae. And the inflammation grading result shown that LGS (25, 50, 100 μg/mL) or TWS119 (30 μM) markedly decreased the larvae’s inflammation grading compared with the LPS group (Figure 5D). However, the zebrafish accepted Wortmannin (LPS + Wortmannin) exhibited bulk adhesion of neutrophils, and few neutrophils remaining or some necrosis were observed in some larvaes yolk. Meanwhile, the reduction of neutrophil inflammatory infiltration and inflammation grading by LGS (100 μg/mL) was prevented by dealing with Wortmannin (Figures 5B–D). The LGS-dependent decrease in GSK3b mRNA (Figure 5E) was also reflected at the protein level. Our results shown that LGS could significantly enhance the expression of p-GSK-3β protein and inhibit GSK-3β expression, while Wortmannin could partially reverse these effects of LGS (Figure 5F).

### LGS inhibits GSK-3β expression in LPS-induced macrophage inflammation

**FIGURE 6:**
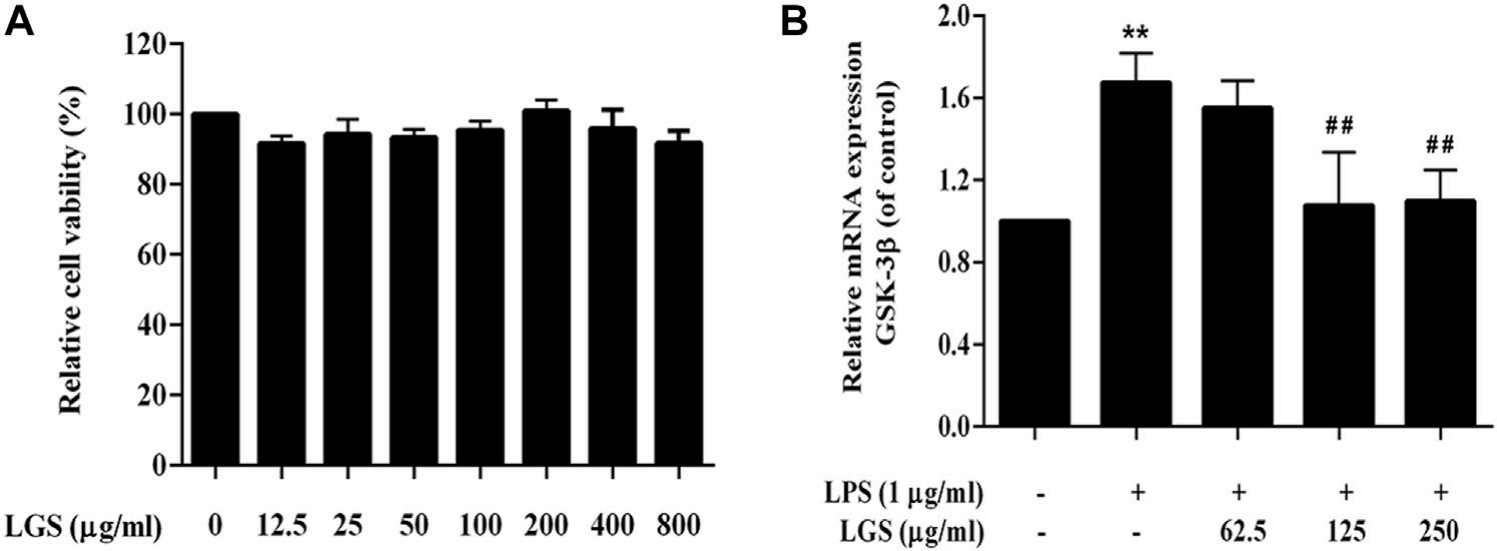
LGS depressed the expression of GSK-3β in LPS-induced macrophage inflammation. **(A)** LGS (12.5–800 μg/mL) showed no significant cytotoxicity in RAW264.7 macrophages. Cell survival was detected by MTT assay. **(B)** Comparison of GSK-3β expression in different treatment groups (LPS group vs. blank group, ^**^ *p* < 0.005; vs. LGS group, ^##^ *p* < 0.005, ^#^ *p* < 0.05).

Similar to neutrophil, activation of macrophages is one of the pathological hallmarks of the inflammatory response (Rittirsch et al., 2008). Studies have proved that GSK-3 is also involved in the macrophage activation in LPS-induced inflammatory response. As showed in Figure 6A, LGS at 12.5–800 μg/mL had no significant cytotoxicity on RAW264.7 cells. In non-toxic doses, LGS treatment groups could significantly reduce the mRNA level of GSK-3β that upregulated by LPS (Figure 6B). It illustrated that LGS could inhibit the expression of GSK-3β in LPS-induced macrophage inflammation.

### LGS inhibits the M1 polarization by downregulated GSK-3β in macrophages

Macrophages are categorized into two types based on their functions: classically activated M1 and alternatively activated M2 macrophages. Under the stimulation of LPS, the total number of macrophages would increase, mainly the proinflammatory M1 and their products, while the anti-inflammatory M2 and their products will decrease correspondingly. This process is regulated by GSK-3β (Jiang et al., 2019).

**FIGURE 7:**
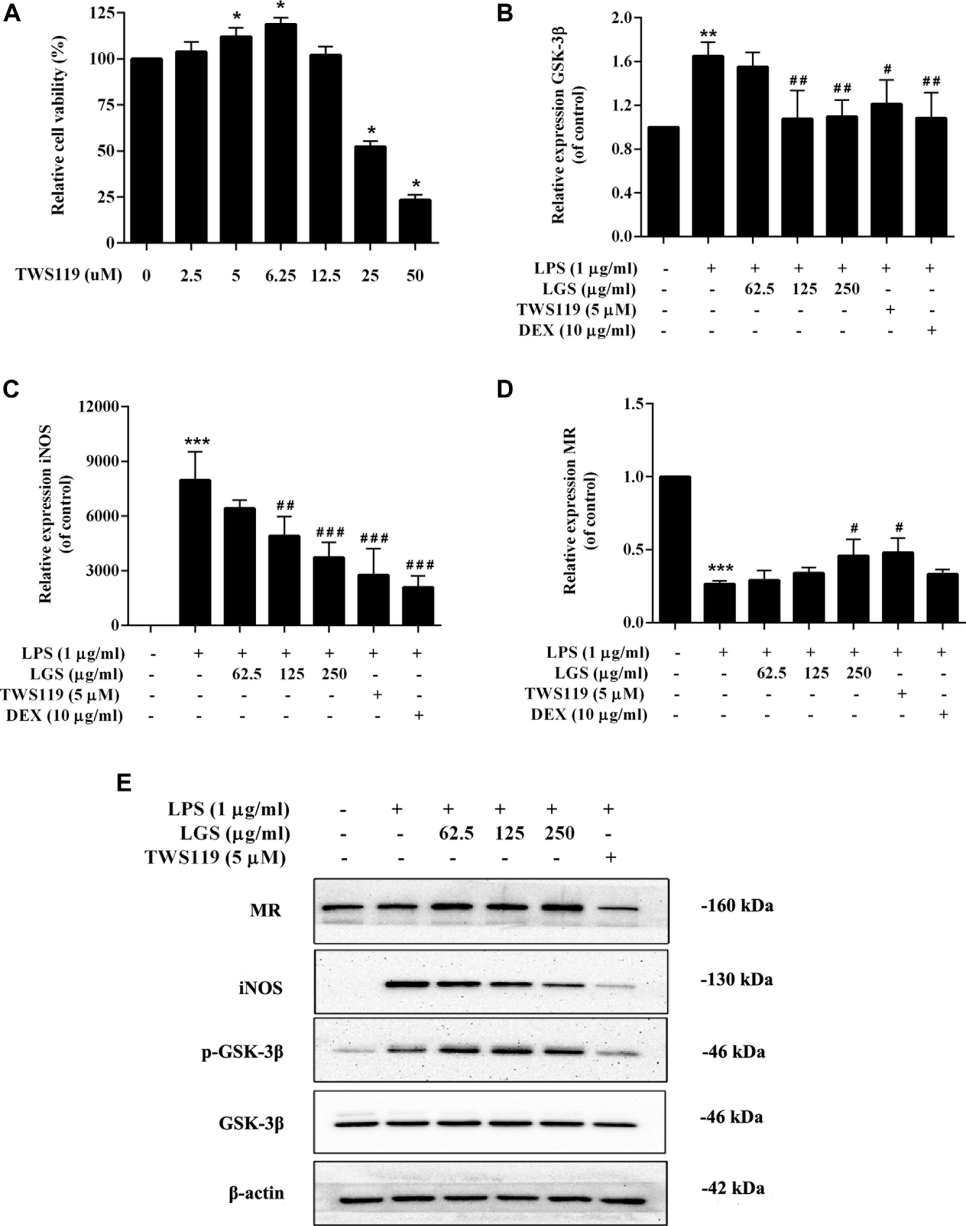
Effect of LGS on LPS-induced macrophage cell polarization. RAW264.7 cells (M0) were pre-stimulated with 100 ng/mL LPS for 48 h to obtain M1 macrophages. LGS (62.5, 125, 250 μg/mL) or TWS119 (5 μM) was co-stimulated with LPS (1 μg/mL) for 48 h. TWS119 (2.5–12.5 μg/mL) showed no significant cytotoxicity in RAW264.7. Cell survival was detected by MTT assay **(A)**, ^*^ *p* < 0.05, vs. blank group). The mRNA expression level of GSK-3β, iNOS and MR were determined by q-PCR **(B–D)**. The protein expressions of iNOS, MR, p-GSK-3β (Ser9) and GSK-3β were measured by Western blot **(E)**. Data are presented as mean ± SEM, n = 3, *p < 0.05, ^***^ *p* < 0.0001, LGS group vs. blank group; ^#^ *p* < 0.05, ^##^ *p* < 0.001, ^###^ *p* < 0.0001, vs. LGS group.

The M1 polarization marker iNOS and the M2 polarization marker Mannose receptor (MR) were tested in RAW264.7 cells along with the inhibitor TWS119 to specifically block GSK-3β activation in order to determine whether LGS affects macrophage polarization via reducing GSK-3β signaling. We found that the non-toxic dose of LGS could significantly reduce the expression of GSK-3β upregulated by LPS, and its inhibitory effect was stronger than the TWS119 group (Figures 7A, B). In addition, the upregulated expression of iNOS mRNA induced by LPS also showed the same tendency of depression by LGS (Figure 7C). In contrast, the expression of MR was significantly downregulated in LPS-stimulated macrophages, and LGS or TWS119 could upregulated the MR mRNA levels (Figure 7D).

WB results showed that LGS could inhibit the LPS-induced upregulation of iNOS in a dose-dependent manner. Meanwhile, LGS could significantly enhance the expression of MR and the phosphorylation of GSK-3β in a concentration-dependent manner. These results suggest that LGS inhibits the M1 macrophages polarization by upregulated the phosphorylation of GSK-3β (Figure 7E).

## Discussion

In recent years, molecular research and bioinformatics techniques have developed rapidly. The application of bioinformatics techniques to explore the underlying molecular mechanisms of disease has become a key approach for researchers. Machine learning is playing an important role in the detection, diagnosis and exploration of therapeutic measures for diseases, and is also being used to study sepsis (Kim et al., 2020; Peiffer-Smadja et al., 2020). One study identified SLC2A6, C1ORF55, DUSP5 and ROHB as key genes in sepsis (AUC>0.75) by the LASSO method, while SLC2A6 is thought to be positively associated with the level of infiltration of Th1 cells (Li et al., 2021). Five significant genes, including NKG7, SPTA1, FGL2, RGS2, and IFI27, were discovered using an SVM model by Xu et al. (2021). These key genes may serve as possible diagnostic indicators for sepsis-induced ARDS, according to the study. However, these studies only explored data mining of publicly available datasets and did not analyze gene sets with specific functions, such as inflammatory chemotactic genes. With this in mind, in order to further our understanding of the physiopathology and molecular mechanisms of sepsis as well as to identify new pharmacotherapeutic targets for clinical treatment, we developed a thorough and in-depth evaluation system to analyze and co-validate *in vitro* and *in vivo* the inflammatory chemotaxis-related signature genes involved in sepsis patients. In this study, we screened 24 differentially expressed inflammatory chemokine genes (DEGs) and found 14 genes upregulated and 10 genes downregulated. Subsequent GO enrichment analysis showed that all DEGs were primarily associated with cellular responses to chemokines, plasma membrane components and cytokine receptor binding activity, while KEGG enrichment analysis showed an association with chemokine signaling pathways, viral protein-cytokine and cytokine receptor interactions, cytokine-cytokine receptor interactions, human cytomegalovirus infection and Kaposi’s sarcoma-associated herpesvirus infection. The results of the enrichment analysis suggest that these DEGs have an essential regulatory role in the inflammatory chemotaxis pathway, while the corresponding cytokines and factor receptors are indispensable for the achievement of inflammatory chemotaxis. Key machine algorithm and LAASO regression analysis identified 1 HUB gene, confirming GSK-3β as a signature gene associated with inflammatory chemotaxis in sepsis. Our *in vitro* and *in vivo* experiments demonstrated that LGS inhibits LPS-induced inflammation by meddling in GSK-3β phosphorylation inactivation to regulate the leukocyte migration and polarization.

GSK-3β is a serine/threonine protein kinase that affects many cellular processes, including proliferation, metabolic regulation, migration, and signaling pathway transduction. A new cellular function regulated by GSK3β was identified by recent findings showing that GSK3β is a potent regulator of inflammation and cell migration. GSK3β is involved in the innate immune response. Innate immune cells, including macrophages, dendritic cells and neutrophils, are the first line of defense against pathogens that can be activated by GSK3β to release cytokines and chemokines, and promote other immune cells to infiltrate damaged tissues (Beurel et al., 2010; Jope et al., 2017). In this study, the “corrplot” R package was used to analyze the relationship between GSK-3β and immune cells, and the results showed that GSK-3β was positively correlated with neutrophil and monocyte infiltration. Relevant studies have also found that inhibition of GSK3β expression can significantly reduce the release of monocyte proinflammatory cytokines and neutrophil infiltration (Martin et al., 2005; Wang et al., 2014; Jiang et al., 2019). Similar to literature reports, our results revealed that both LGS and TWS119 treatment reduced yoke congestion, inflammatory cell infiltration and improved pathological conditions due to LPS. And this effect of LGS was reversed by the Wortmannin (Figure 5A). These results suggested that LGS reduced inflammatory damage through the inhibition on GSK-3β.

Besides producing molecules associated with the inflammatory response, a closely linked important characteristic of inflammatory cells is their capacity to migrate. Each step of cell migration is regulated by many intracellular signals, one of which is GSK-3β. GSK-3β acts as a downstream involvement with the CKLF1/CCR5 axis to influence neutrophil migration (Jiang et al., 2019). Neutrophil migration plays a crucial role in host multi-site functional impairment induced by septic shock. Studies showed that GSK-3β was inactivated by phosphorylation of Ser9, which could inhibit the activation of neutrophils and decrease LPS-induced endotoxic shock (Dugo et al., 2007; Park et al., 2014; Molagoda et al., 2021). Similar to findings in the literature, our research demonstrated that LGS could decrease LPS-induced neutrophil inflammatory migration and recruitment in zebrafish by suppressing the expression and activity of the GSK-3β (Figures 5B–F). TWS119 had similar outcomes. However, treating with Wortmannin was able to mitigate some of this LGS effect. In the meantime, LGS was able to increase the expression of p-GSK-3β in LPS-induced RAW264.7 cells (Figure 7E). It was suggested that LGS may inactivate GSK-3β by phosphorylation of Ser9, reduce LPS-induced leukocytes migration in sepsis.

Cell migration has been conceptualized as a cyclic process of cell movement initiated by polarization of a cell (Vicente-Manzanares et al., 2005). Macrophages can modulate their polarization into pro-inflammatory M1macrophages or anti-inflammatory M2 types depending on their microenvironment. Based on the function of M2 macrophages to inhibit inflammation and promote tissue repair, many researchers have tried to treat or alleviate diseases by regulating the balance of M1/M2. It was found that GSK-3β involved in the polarization effect of macrophage activation, and inhibition of GSK-3β could promote the transformation of M1 macrophages into M2 macrophages by up-regulating PPARγ activity (Jiang et al., 2016). Our results show that LGS could inhibit the LPS-induced upregulation of GSK-3β and iNOS in a dose-dependent manner. Meanwhile, LGS could significantly enhance the expression of MR. In addition, WB showed that LGS could downregulate iNOS expression in a dose-dependent manner, upregulate the levels of MR and p-GSK-3β-Ser 9. Studies have shown that phosphorylation inactivation of GSK-3β-Ser 9 in mouse models infected with MHV or *E. coli* can lead to a significant increase in GSK3β activity and significant secretion of pro-inflammatory cytokines (Sy et al., 2011). Inducing GSK-3β phosphorylation inactivation inhibits sepsis-induced cardiac dysfunction and mitochondrial dysfunction (Liu Z. et al., 2022). Our findings are similar to those above, suggesting that LGS can exert its anti-inflammatory activity by promoting phosphorylation inactivation of GSK-3β proteins, which in turn promotes the transformation of macrophages M1 to M2.

## Summary and prospect

Bioinformatics studies have shown that GSK-3β is a core target associated with inflammatory chemotaxis in sepsis, and its expression is positively correlated with neutrophil chemotaxis, which is the main anti-inflammatory target of LGS. In addition, *in vitro* and *in vivo* studies have confirmed that LGS can regulate neutrophil migration and macrophage M1/M2 balance by interfering with GSK-3β expression. This is one of the important mechanisms of LGS to reduce sepsis and improve the long-term prognosis of sepsis. Although this work has been validated in cell and animal models, further clinical trials are needed to confirm it.

## Data Availability

The original contributions presented in the study are included in the article/Supplementary Material, further inquiries can be directed to the corresponding author.
