# Supplementary material for: Liang-Ge-San: a classic traditional Chinese medicine formula, attenuates acute inflammation via targeting GSK3β
Source: Front Pharmacol. 2023 Jun 29;14:1181319. doi: 10.3389/fphar.2023.1181319 (PMC10338930; doi:10.3389/fphar.2023.1181319)
Supplement: Supplementary file 1 [file Table1.docx]

**Supplementary Table1.**Functional analysis of DEGs (only the first 10 enrichment items are exhibited).

| **Enrichment Projects** | **ID** | **Description** | **P value** | **P. adjust** | **Q value** | **Count** | **Gene Ratio** |
| --- | --- | --- | --- | --- | --- | --- | --- |
| GO-BP | GO:0070098 | chemokine-mediated signaling pathway | 3.07E-20 | 4.29E-17 | 2.06E-17 | 11 | 11/24 |
| GO-BP | GO:1990868 | response to chemokine | 9.49E-20 | 4.42E-17 | 2.12E-17 | 11 | 11/24 |
| GO-BP | GO:1990869 | cellular response to chemokine | 9.49E-20 | 4.42E-17 | 2.12E-17 | 11 | 11/24 |
| GO-BP | GO:0060326 | cell chemotaxis | 1.47E-19 | 5.12E-17 | 2.46E-17 | 14 | 14/24 |
| GO-BP | GO:0019221 | cytokine-mediated signaling pathway | 5.39E-17 | 1.51E-14 | 7.22E-15 | 14 | 14/24 |
| GO-BP | GO:0050900 | leukocyte migration | 1.13E-16 | 2.62E-14 | 1.26E-14 | 13 | 13/24 |
| GO-BP | GO:0030595 | leukocyte chemotaxis | 1.64E-15 | 3.27E-13 | 1.57E-13 | 11 | 11/24 |
| GO-BP | GO:0071674 | mononuclear cell migration | 2.17E-14 | 3.78E-12 | 1.81E-12 | 10 | 10/24 |
| GO-BP | GO:0097529 | myeloid leukocyte migration | 6.94E-14 | 1.08E-11 | 5.16E-12 | 10 | 10/24 |
| GO-BP | GO:0071675 | regulation of mononuclear cell migration | 1.07E-12 | 1.50E-10 | 7.18E-11 | 8 | 8/24 |
| GO-CC | GO:0009897 | external side of plasma membrane | 2.35E-08 | 1.79E-06 | 1.07E-06 | 8 | 8/24 |
| GO-CC | GO:0031234 | extrinsic component of cytoplasmic side of plasma membrane | 6.59E-06 | 0.000250248 | 0.00014904 | 4 | 4/24 |
| GO-CC | GO:0019898 | extrinsic component of membrane | 3.17E-05 | 0.000803445 | 0.000478506 | 5 | 5/24 |
| GO-CC | GO:0009898 | cytoplasmic side of plasma membrane | 5.36E-05 | 0.00085188 | 0.000507352 | 4 | 4/24 |
| GO-CC | GO:0019897 | extrinsic component of plasma membrane | 5.60E-05 | 0.00085188 | 0.000507352 | 4 | 4/24 |
| GO-CC | GO:0098562 | cytoplasmic side of membrane | 9.07E-05 | 0.00114933 | 0.000684504 | 4 | 4/24 |
| GO-CC | GO:0005911 | cell-cell junction | 0.002864023 | 0.031095106 | 0.018519246 | 4 | 4/24 |
| GO-CC | GO:0005901 | caveola | 0.004735072 | 0.041860923 | 0.024931021 | 2 | 2/24 |
| GO-CC | GO:0005758 | mitochondrial intermembrane space | 0.004957215 | 0.041860923 | 0.024931021 | 2 | 2/24 |
| GO-CC | GO:0031970 | organelle envelope lumen | 0.006138636 | 0.046302885 | 0.02757651 | 2 | 2/24 |
| GO-MF | GO:0016493 | C-C chemokine receptor activity | 5.99E-16 | 4.73E-14 | 1.77E-14 | 7 | 7/24 |
| GO-MF | GO:0019957 | C-C chemokine binding | 8.45E-16 | 4.73E-14 | 1.77E-14 | 7 | 7/24 |
| GO-MF | GO:0001637 | G protein-coupled chemoattractant receptor activity | 1.60E-15 | 4.73E-14 | 1.77E-14 | 7 | 7/24 |
| GO-MF | GO:0004950 | chemokine receptor activity | 1.60E-15 | 4.73E-14 | 1.77E-14 | 7 | 7/224 |
| GO-MF | GO:0019956 | chemokine binding | 1.04E-14 | 2.44E-13 | 9.16E-14 | 7 | 7/24 |
| GO-MF | GO:0004715 | non-membrane spanning protein tyrosine kinase activity | 1.99E-11 | 3.91E-10 | 1.47E-10 | 6 | 6/24 |
| GO-MF | GO:0004896 | cytokine receptor activity | 2.96E-11 | 4.98E-10 | 1.87E-10 | 7 | 7/24 |
| GO-MF | GO:0042379 | chemokine receptor binding | 3.73E-10 | 4.97E-09 | 1.86E-09 | 6 | 6/24 |
| GO-MF | GO:0019955 | cytokine binding | 3.79E-10 | 4.97E-09 | 1.86E-09 | 7 | 7/24 |
| GO-MF | GO:0140375 | immune receptor activity | 4.87E-10 | 5.74E-09 | 2.15E-09 | 7 | 7/24 |
| KEGG | hsa04062 | Chemokine signaling pathway | 1.62E-40 | 2.68E-38 | 1.56E-38 | 24 | 24/24 |
| KEGG | hsa04061 | Viral protein interaction with cytokine and cytokine receptor | 1.08E-15 | 8.94E-14 | 5.19E-14 | 11 | 11/24 |
| KEGG | hsa04060 | Cytokine-cytokine receptor interaction | 6.77E-12 | 3.72E-10 | 2.16E-10 | 12 | 12/24 |
| KEGG | hsa05163 | Human cytomegalovirus infection | 6.76E-09 | 2.79E-07 | 1.62E-07 | 9 | 9/24 |
| KEGG | hsa05167 | Kaposi sarcoma-associated herpesvirus infection | 4.45E-08 | 1.47E-06 | 8.53E-07 | 8 | 8/24 |
| KEGG | hsa04935 | Growth hormone synthesis, secretion and action | 2.08E-05 | 0.000572241 | 0.00033221 | 5 | 5/24 |
| KEGG | hsa04917 | Prolactin signaling pathway | 4.51E-05 | 0.001064207 | 0.000617817 | 4 | 4/24 |
| KEGG | hsa04666 | Fc gamma R-mediated phagocytosis | 0.000161846 | 0.003338081 | 0.001937897 | 4 | 4/224 |
| KEGG | hsa04933 | AGE-RAGE signaling pathway in diabetic complications | 0.000182094 | 0.003338395 | 0.00193808 | 4 | 4/224 |
| KEGG | hsa04620 | Toll-like receptor signaling pathway | 0.000211866 | 0.003495793 | 0.002029455 | 4 | 4/24 |
