# Supplementary material for: Liang-Ge-San: a classic traditional Chinese medicine formula, attenuates acute inflammation via targeting GSK3β
Source: Front Pharmacol. 2023 Jun 29;14:1181319. doi: 10.3389/fphar.2023.1181319 (PMC10338930; doi:10.3389/fphar.2023.1181319)
Supplement: Supplementary file 2 [file Table2.docx]

**Supplementary Table2.** Feature genes obtained by different machine learning algorithms.

| Algorithms | Count | Gene Name |
| --- | --- | --- |
| SVM-REF | 20 | GSK3B/CCL5/GNG5/LYN/HCK/CCL4/STAT5B/CCR7/PXN/CCR1/ITK/CCR3/CX3CR1/PIK3CB/CXCL16/JAK2/CXCL5/CCR6/ADCY3/PRKCD |
| LASSO | 12 | ITK/GSK3B/CCR6/CCR9/GNG5/CCL5/CCR3/CXCL5/CX3CR1/CCR1/CCR7/CCL4 |
| RF | 12 | GSK3B/ITK/CCR6/CCL5/STAT5B/PIK3CB/CX3CR1/ADCY3/GNG5/CCR9/FGR/CCR3 |
